# ‘I’m not just being difficult . . . I’m finding it difficult’: A qualitative approach to understanding experiences of autistic parents when interacting with statutory services regarding their autistic child

**DOI:** 10.1177/13623613231212794

**Published:** 2023-11-24

**Authors:** Sarah Radev, Megan Freeth, Andrew R Thompson

**Affiliations:** 1 Cardiff University, UK; 2 University of Sheffield, UK

**Keywords:** adults, advocacy, autism spectrum disorders, education services, health services, parents, qualitative research, schoolage children

## Abstract

The experiences of autistic parents when interacting with statutory services for their autistic children is not well understood. This study investigates the experiences of autistic parents’ interactions with statutory services, including education and healthcare, in relation to their autistic children. Interpretative Phenomenological Analysis was used to investigate their experiences. Ten autistic mothers of autistic children in mainstream education completed semi-structured interviews, which were co-developed with autistic parents. Two superordinate themes were developed: (1) The wider system is the problem and (2) Feeling judged and stigmatised. While Theme 1 demonstrates some similarities in the experiences of autistic parents to that of non-autistic parents in previous studies, theme 2 is unique to autistic parents who may feel that they are being treated differently. These themes indicate the importance of training encouraging a strength-based rather than deficit-based understanding of autism. Participants highlighted the wider system is the problem rather than individual professionals, and that it places some families at a disadvantage.

**Lay abstract:**

Becoming a parent is an important part of adult life for many people, including autistic people. Many parents of autistic children can find getting the right support for their children difficult. Knowledge is currently poor about how this is experienced by parents who are also autistic themselves. The main researcher is also an autistic parent to an autistic child and other experts by experience were consulted in the development of the study. Ten autistic mothers with autistic children in mainstream education were interviewed about their experiences of seeking support for their autistic children from services such as healthcare and education. Participants talked about finding the overall system being the main problem, rather than the individuals working in it, and about needing to fight to get the right support for their children. These are points that non-autistic parents have also raised before. Participants also talked about feeling judged and stigmatised for being autistic, and about struggling to manage sensory and communication difficulties, which is something that has not been talked about by non-autistic parents. Improving services to offer better support to autistic families is important and can be achieved through better training. This training should be developed and run by autistic adults and focus on positive aspects of autism, rather than negative.

## Introduction

Parenthood is an important part of adult life for many, bringing with it new challenges, responsibilities, celebrations and disappointments. Experiences of parenthood can have a direct impact on a person’s well-being, both positively and negatively (Nelson et al., 2013; Umberson et al., 2010), and yet parenthood is an area of adult life that has been particularly neglected in autism research. To date, the extensive research on autism and parenthood has largely focused on non-autistic parents of autistic children (Boshoff et al., 2016; Celia et al., 2020; Chang et al., 2019; Sproston et al., 2017), rather than on autistic adults as parents themselves of autistic children. Boshoff et al. (2016) conducted a meta-synthesis of parents’ experiences of advocating for their autistic children while other articles have focused on perspectives of school exclusion (Sproston et al., 2017) and the lived experience of caring for an autistic child (Celia et al., 2020). Existing research indicates that parents of autistic children are likely to experience greater levels of stress, not only when compared to parents of typically developing children, but also when compared to parents of children with other disabilities (Boshoff et al., 2016; Burke & Hodapp, 2014; Chang et al., 2019; Lau & Peterson, 2011).

Acting as an advocate in the context of being a parent was defined by Boshoff et al. (2016) as obtaining support or service for a child, promoting their wellbeing and rights, being their voice and educating others. The act of advocating for an autistic child is something many parents find necessary during the process of seeking a diagnosis, and ongoing support in health services and educational settings (Burke & Hodapp, 2014; Sedgewick et al., 2018). Burke and Hodapp (2014) identified a strong correlation between positive parent–school relationships and reduced maternal stress and found that where parents felt the need to engage in anything more than few or no advocacy activities, due to feeling dissatisfied with the support received, increased stress levels were recorded. This highlights the importance of positive relationships with schools. Such advocacy can be extremely emotionally charged, involve conflicts arising with professionals and requiring a certain level of agency and self-efficacy on the part of the parents (Boshoff et al., 2016). This form of advocating is clearly challenging and emotive, and has been described by parents as being a ‘battle’ and a ‘fight’ (Celia et al., 2020; Marriott et al., 2022; Sedgewick et al., 2018; Sproston et al., 2017).

It is especially important to consider the experiences of autistic parents interacting with services on behalf of their autistic children, to expand on the existing literature on non-autistic parents, as genetics can play a strong role in the likelihood of autism in a family (Sandin et al., 2014; Taylor et al., 2021; Tick et al., 2016). Although experiences of autistic parents advocating for their children has been touched on in subthemes of articles with a wider focus, to date, there has not been focused investigation of how interactions with statutory services are experienced by autistic parents. The SPACE framework laid out by Doherty et al. (2023) recommends attention is paid to sensory needs, predictability, acceptance, communication and empathy when meeting the needs of autistic people, which would be just as applicable to autistic parents supporting their children, as to autistic people who are being supported directly.

Autistic parents often feel that they are more knowledgeable about autism than the professionals (Crane et al., 2021), but may not find their concerns are taken seriously or feel misunderstood (Dugdale et al., 2021). Others have raised concerns about stigma relating to their own diagnosis, which leads to a reluctance to disclose to professionals for fear of judgement and being deemed unable to cope (Bertilsdotter Rosqvist & Lövgren, 2013; Dugdale et al., 2021; Marriott et al., 2022; Pohl et al., 2020). Previous research has indicated perceived stigma from others may contribute to poorer mental health and increased sense of social isolation in autistic individuals. This may also lead to an increase in tendencies to engage in ‘masking’ or ‘camouflaging’ (Cage et al., 2018). It is therefore important to investigate the lived experience of autistic parents when interacting with statutory services on behalf of their autistic children to better understand the needs of autistic individuals and ensure that appropriate support is provided for autistic families.

## Methods

### Methodological approach

This study employed a qualitative approach using Interpretative Phenomenological Analysis (IPA), which involves a double-hermeneutic process whereby the experiences of the participant are first interpreted and made sense of by themselves through the process of recounting their experiences during the interview, and second interpreted and made sense of by the researcher during the analysis process (Larkin & Thompson, 2012; Larkin et al., 2006; Smith & Nizza, 2022). Semi-structured interviews were identified as the most appropriate means of gathering information from participants about their experiences. Ethical approval for this study was granted by the Cardiff University School of Psychology Research Ethics Committee.

### Community involvement

The first author is herself an autistic parent of an autistic child and so had some insight into the primary issues when developing the semi-structured interview questions and recruitment materials. A reflective log was kept throughout the process to ensure that the first author could reflect and utilise personal and theoretical knowledge without personal experiences of advocating for support in education and healthcare biasing the interpretations. Two other autistic parents were recruited through social media prior to recruitment of interview participants to provide feedback on the information provided and proposed semi-structured interview questions. Their suggestions resulted in additional prompts being added to the semi-structured interview schedule ensuring a wider perspective than just that experienced by the lead author, and the removal of the term ‘high-functioning autism’ in relation to participant inclusion criteria from the recruitment documentation. This is due to terms such ‘high’ and ‘low’ functioning being outdated and linked to a linear understanding of the autism spectrum. which many in the autism community can find unhelpful.

### Participants

Ten participants, all of whom were women, were recruited via purposive sampling on social media sites and through the Autistica research database. The first author disclosed their own diagnosis to participants at the point of recruitment and several participants expressed that this strengthened their interest in being involved as they trusted the interviewer would understand their experiences and represent them sensitively. It is likely that this resulted in more open disclosures of experiences than may otherwise have been achieved with a non-autistic interviewer. Participants were identified as either meeting or not meeting inclusion criteria via completing an online survey. Inclusion criteria were: over 18 years of age, fluent in English, formal diagnosis of autism, parent of an autistic child currently attending mainstream school. Participants were excluded if they were not resident in the United Kingdom; their autistic child was not in mainstream school; they had a co-occurring intellectual disability (ID), which would have made it difficult to know if experiences were impacted by being autistic, or due to having an ID. These exclusions were also intended to ensure a homogeneous sample, which is in keeping with an IPA approach. See Table 1 for demographic information.

**Table 1.** Demographic information of participants.

Category	No. of participants
Age range	(mean 46.6)
35–40	2
40–45	1
45–50	3
50–55	4
Time since diagnosis (years)	(mean 2.9)
< 1	4
1–2	2
4–5	3
> 5	1
Ages of autistic child/children (years)	
5–10	3
11–17	12
Ethnicity	
White British	8
Mixed race	2
Further education	
NVQ	1
Undergraduate degrees	4
Masters	1
PhD	3
Employment status	
Student	1
Unemployed	3
Employed	6
Marital status	
Married/Co-habiting with child’s father	8
Co-parenting with child’s father	1
Single parent	1

NVQ: national vocational qualification.

### Procedure

Semi-structured interviews were conducted by the first author, two via telephone calls and eight via online video call as per participants’ preferences. The primary interview schedule (see Table 2) was developed to illicit the sharing of lived experiences of different statutory services, such as healthcare, school and social services in a way that did not lead interviewees in a particular direction. This was particularly important given the interviewers own personal experiences of interacting with services as an autistic parent of an autistic child. Questions were designed to be simply open invitations to talk about experiences with the relevant services. Informed consent was obtained prior to the interviews commencing. Interviews ranged in length from 48 to 92 min (mean 63.4 min) and were audio recorded and transcribed verbatim. Throughout the interview process, the lead researcher clarified points made by participants to ensure an accurate representation of their experiences and to avoid potential biases based on personal experiences. Participants were de-briefed at the end of the interview and given the opportunity to talk through anything they found upsetting when recounting difficult experiences, and to share with them the planned outcomes of the research.

**Table 2.** Primary questions asked within the semi-structured interview.

	Questions
1.	What healthcare professionals have been involved in supporting your child?
2.	Does your child receive any support from Social Services?
3.	Can you tell me about how things have been with your child’s school?
4.	What have you shared or talked about in relation to your own diagnosis to professionals when talking to them about your child?
5.	How have your experiences of interacting with professionals regarding your autistic child impacted on your experience of being a parent?
6.	What are your greatest sources of support in this area of your life?
7.	Is there anything else that you have not had the chance to tell me about today that you feel would be important for me to know about your experience of being an autistic parent seeking support for an autistic child?

### Analysis

**Figure 1.**
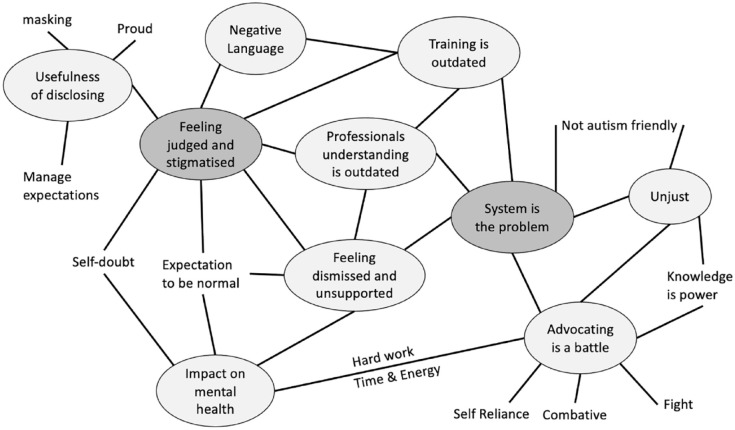
Conceptual mapping example.

Prior to formal analysis, transcripts were read closely while re-listening to the audio to ensure that the full context of participant responses was considered. The IPA analysis process outlined by Larkin and Thompson (2012), Smith et al. (2009) and Smith and Nizza (2022) was adopted and began with careful analysis of each interview transcript to identify meanings and experiential themes. Exploratory notes were made on descriptive, linguistic and conceptual elements line-by-line in the right-hand margin of the transcript. The researcher then engaged in interpretative coding to develop experiential themes, which were noted in the left-hand margin, linked to supporting quotes and compiled to create overarching themes. This process was then repeated with each transcript on an individual basis before a cross-case interpretative analysis was conducted identifying overlapping patterns and themes through conceptual mapping (see Figure 1). This was then compiled into a table of superordinate themes relevant to the studies aims (Smith, 2004).

The process of data collection and analysis conducted by the first author was audited using a checklist to verify and evidence each step during consultation with the last author.

## Results

During the cross-case analysis, two superordinate themes came through as particularly important, each of which had further subthemes. These themes are outlined in Table 3. Pseudonyms have been used to protect the anonymity of participants.

**Table 3.** Themes.

Superordinate theme	Subtheme
The wider system is the problem	Feeling dismissed and unsupported
	The system is unjust Need to fight for the right support
Feeling judged and stigmatised	Use of negative language, which is offensive and misleading
	Training of professionals is not good enough
	Usefulness of disclosing diagnosis

### Theme 1 – the wider system is the problem

All participants felt the wider system is a greater problem than the professionals working in it. Most talked about experiences of feeling dismissed and unsupported and how the system being unjust is particularly difficult to manage as an autistic person.

#### Feeling dismissed and unsupported

Participants experienced a lack of support from statutory services and even that they ‘don’t get a lot of support from anywhere really’ (Rachel). Participants used words such as ‘fobbed off’ (Kayleigh, Tara), ‘rejected’ (Kim) and ‘dismissive/dismissed’ (Susan, Kim, Elaine, Dawn) and felt they needed to rely on themselves rather than professionals and services due to the lack of support received.

Some talked about lack of consistency and communication between services who ‘don’t share information’ (Chloe) with each other, which results in being passed back and forth between services instead of being offered support from the beginning:

So we end up in this situation where we can try and get support from places like Social Services, and they just pass us back and forth between teams, blaming the other entity . . . saying, ‘Well, they need to deal with it’. (Kim)

Such experiences led to a feeling among participants that there was a lack of responsibility taken for providing support by services, placing the onus back on the parents. However, there was also an acknowledgement from most participants that the individual professionals they encountered had the best intentions and argued it’s ‘not [their] fault’ (Chloe) and they are ‘doing the best they can’ (Claire). Kim commented that ‘the vast majority of healthcare professionals are trying their best . . . most have wanted to help but can’t’. Rather than the problem lying in individual professionals, participants very much felt that the problem lies in the way the system is set up:

I think I am frustrated with the systems and processes more than with the people . . . And I can understand that people are forced to work within systems that they haven’t designed. (Claire)

Some participants talked about feeling as though professionals in services have been ‘lying’ (Elaine) to them where promises to provide support were given and then not followed through, which left them feeling misled and let down. Participants commented on there having been a ‘breakdown’ (Kelly) in that professionals ‘promise you all these things . . . that never actually come to fruition’ (Dawn). This lack of support and the need for self-reliance has an impact on their own mental health and sense of trust, not only in the systems, but also in themselves, having a negative impact on their self-confidence.

Actually, they were very dismissive and made me feel very stupid and as if I was just an anxious person making some stuff up . . . it really has impacted on me and made me feel . . . inadequate . . . and foolish. (Susan)

For some participants, this feeling of being unsupported had a direct impact on their experience of parenting causing them to feel like a ‘rubbish mum because I feel like we didn’t have enough fun’ (Kim) and that there is ‘much less space for creativity and spontaneity and just joyous stuff’ (Elaine) with time being spent on navigating systems to get the right support and delivering therapy at home, rather than on family life.

#### The system is unjust

Participants expressed a sense of injustice at not getting the support they were promised and entitled to. This is in keeping with the rigid thinking patterns, which are common in autistic individuals (American Psychiatric Association, 2013) and was felt particularly keenly when the specific rules laid down in legislation were not being followed:

I found that process really distressing, as any mum would, wanting the best for your child, but also as a quite pedantic autistic person with this massive sense of injustice, because I was like, ‘They’re not doing this by the rules. They are breaking them’. (Kim)

The systems were also described as being ‘non-accessible to an autistic person’ (Elaine) and ‘extremely ableist’ (Chloe) where there was a requirement to travel long distances to medical appointments despite not having a car, and an expectation that the parent is able to fill out lengthy assessment forms by hand without support. The need for equality, justice and things being done a certain way being a particularly autistic trait was brought up by several participants. One participant commented in relation to education not providing support outlined in the special educational needs (SEN) guidelines due to a lack of funding, ‘I think an Autistic persons’ understanding of what your entitlements are was like, “Well I’m entitled to that I shouldn’t have to fight for it”’ (Elaine).

The result of this is an awareness of being viewed as a pain by professionals. One participant commented with regards to communication with their child’s school about adjustments to the uniform requirement due to sensory needs ‘they just see me being a pain. Demanding reasonable adjustments, which actually are within the law that they need to do, and I have said that to them’ (Kelly). Feeling like a ‘pain’ in this way may have a negative impact on parental relationships with services, which may be detrimental to the support the child receives.

There was also a frustration expressed regarding the unjust inequality of the system, which does not provide support equally to all children, but only to those able or willing to fight, or to invest money. One participant felt that ‘the parents who shout the loudest or have some bit of money to spend, they often get the more support, but that’s actually not very fair for people who haven’t’ (Tara) when reflecting on her own privileged position to be able to get the support for her son, where others may struggle more. This inequality in the system is another way the overarching system has been experienced as the problem, rather than the individuals within the system.

#### Need to fight for the right support

Feeling unsupported in a wider system that often feels unjust also left participants feeling that they needed to fight for the right support. Participants referred to the process of advocating as ‘a minefield to navigate’ (Dawn) and needing to ‘fight and scream and shout every step of the way’ (Elaine).

Participants talked about the need to ‘save my own sanity’ (Dawn) and feeling a sense of ‘desperation’ (Kelly). The emotional impact was wide reaching and for some had impacted on all areas of their life: ‘I think that the combination of the battles with the SEN system and school, getting older . . . just meant there was just a total breakdown in my mental health.’ (Elaine).

There was a real sense of this battle being a never-ending tiring experience, described as mentally ‘exhausting’ (Dawn) in terms of the constant thinking about what needs to be done to achieve a particular goal, and the difficulty with picking which battles to fight resulting in ‘limited energy’ (Susan). Participants all talked about numerous interactions with services, which had felt like a battle from getting the diagnosis to receiving appropriate therapeutic support and ongoing support in education. For some, this had involved going to tribunal to get the support they wanted (Kayleigh, Kim). These multiple battles were described by one participant as ‘like you’re running on the treadmill, and it will never stop’ (Kayleigh) and it was highlighted that this can have a negative impact on sleep, wellbeing and a family’s social life.

This level of exhaustion was also linked to a reduced ability to advocate effectively for their children and a sense it was important to prioritise certain battles over others:

I have limited energy to try and pick the battles that are the most important so that I don’t burn all the bridges and damage all the relationships with the very people that are in the position of supporting my children. (Susan)

Participants also talked about how the struggle they experience with managing this battle can be misunderstood by professionals as them being difficult: ‘I *am* an autistic adult trying to navigate this system to advocate for my autistic children. I’m not just being difficult . . . I’m *finding* it difficult’ (Kelly).

Several participants had a positive narrative regarding how being autistic enabled them to use knowledge, describing themselves as ‘focused’ (Tara); using ‘determination and my autistic features’ (Kayleigh); and being a ‘typical autistic person [who] did lots of research’ (Kelly). One participant commented ‘forewarned is forearmed, so to speak isn’t it . . . it’s knowledge that breaks down all the barriers for everything’ (Chloe). In this respect, participants demonstrated how in some ways they perceived themselves to be at an advantage being autistic, because they were able to apply themselves to the fight.

### Theme 2 – feeling judged and stigmatised

Participants talked about feeling judged and stigmatised through the language used indicating outdated and stereotyped understandings resulting from poor autism training. There were mixed views among participants on whether it was beneficial or detrimental to disclose their own diagnosis.

#### Use of negative language offensive and misleading

One way participants felt they were being judged and stigmatised was through the use of ‘negative language’ (Chloe) and outdated terminology such as ‘high’ or ‘low functioning’, to describe autism, which can misrepresent the actual level of need: ‘it’s the classic sort of thing why we don’t use the functioning language anymore. “Oh he’s high functioning” and then *totally* disrespect his needs’ (Elaine).

Functioning language was thought to be problematic because describing a person as either high or low functioning comes with the implication a person can be more or less autistic, which undermines the needs and experiences of those classed as ‘high functioning’ (Rachel) and leads to the view from professionals that ‘well you know, they’re functioning’ (Chloe) and ‘ingrained expectations’ (Elaine) on what support needs the child does or does not have:

The doctor that we were trying to see . . . basically drew a line and was talking about how you can be more or less autistic, and I knew immediately that wasn’t right. (Kelly)

Some participants talked about how ‘ridiculously deficit based’ (Susan) and potentially offensive the language used can be rather than there being a focus on strengths, and the importance of being ‘disability positive [and] neurodiversity positive’ (Kim) in the language used:

I registered a complaint, and the manager of the service phoned me up, and began talking about autistic spectrum disorder to me. Oh my God! I really think that that’s quite an offensive term now, because I’m not disordered, and Jack isn’t disordered. (Claire)

Indeed, the language a society uses can be extremely powerful in shaping understandings and the point was raised by one participant that with the negative connotations of the language used, comes a reluctance from professionals to even raise the possibility of autism with parents:

They’re just so reluctant to use that label. You know because it’s almost as if autism is this stain on people’s record . . . They don’t want to use it . . . it’s just a type of person, it’s not anything to be scared of . . . But they’re making people scared with the negative language. (Chloe)

This raises the concern that the language used may result in a diagnosis being received not only by parents, but by society, as something negative, stigmatising and even something to be ‘scared of’. Deficit-based language may also impact on perceptions of support needs of the children, and ultimately lead to wrong, or inadequate support being provided. However, it is positive to note that things may already be moving in the right direction as one participant also reflected on experiences of the language used being much more positive and strength focused.

I even noticed when my younger two were diagnosed that the way the diagnosis was put to me was much more positive. They were using much more positive language . . . This is a strength . . . this could be a strength of your child’s . . . (Chloe)

#### Training is not good enough

Training provided to professionals in general, across both healthcare and education settings was described as ‘absolutely awful’ (Chloe), ‘out of date’ (Elaine, Susan) and often the lack of understanding demonstrated through the language being used was attributed to professionals not having adequate training in autism.

One of the impacts of professionals not having adequate training was the pressure it placed on parents to then take on the responsibility of ‘doing that advocacy and that educating alongside’ (Kelly) and to ‘upskill and train’ (Susan) the professionals themselves:

If the teachers had a lot more training and understanding of all the different additional needs, they would be able to pick them up quicker and . . . the parents wouldn’t have to. (Tara)

It was also felt training was too much based on outdated and medicalised models of autism, which results in ‘quite a rigid view’ (Kim), ‘stereotypical attitudes’ (Elaine) and that there was a need for professionals to ‘think of the social model a little bit more, within training’ (Claire).

Despite experiences of feeling that professionals did not have adequate training, many participants had the experience that those with personal experience of autism demonstrated the best understanding, and described them as ‘really understanding’ (Rachel) and ‘really helpful’ (Dawn). One participant recounted how important the support of one teacher in the school playground had been to her: ‘she made a point of going over to me and explaining what everyone was doing . . . And she just . . . knew that I would need help with that. It wasn’t judgemental’ (Chloe).

#### Usefulness of disclosing diagnosis

Participants had mixed views on the usefulness of disclosing their own diagnosis due to the mixed responses from professionals with some feeling it was an important part of spreading awareness and being listened to while others were fearful of being judged as people would ‘use it against me’ (Tara):

There is a worry as an autistic parent, because there is a lot of stigma there, that if you say that you’re struggling, that can be a lot more detrimental than it can be for other parents. (Kim)

Some who had disclosed experienced a reaction indicating professionals had changed their view of them, and were somehow viewing them as less capable because of the diagnosis. One participant shared ‘I’ve disclosed to some people in situations that I am also autistic. And then you almost get that sort of patronising when they start to speak more slowly’ (Kelly).

In contrast, many participants had positive experiences when disclosing as they felt that doing so gave them a lot more credibility and helped them feel ‘empowered’ (Chloe) and placed them in a position where they could help educate about autism: ‘I think being an autistic parent with an autistic child has made me much more credible . . . and I think it’s been harder for people to fob us off’ (Kayleigh).

Another experience that came up for several participants was the awareness professionals were assuming they were not themselves autistic. The understanding that ‘autistic children have autistic parents is not taken seriously’ (Claire). Feeling that the same level of communication was expected from autistic parents as non-autistic parents was a source of frustration and participants expressed that it was most helpful when professionals understood that the parents may themselves be autistic: ‘working on the assumption that one or other of the parents is likely to be on the spectrum is probably a reasonable place to start’ (Elaine).

One participant reflected on school concerts being set up in a way that supports the additional needs of any children involved, but the impact of the noise and lighting on autistic parents in the audience is not considered, necessitating sitting somewhere near an exit to easily escape if it becomes too overwhelming.

I think that schools forget . . . that autistic children come from autistic parents generally on the whole. And they put stuff in place for the kids, but then don’t think about how that’s going to affect their parents. (Kelly)

The inconsistency in the experiences and views on disclosure suggests variability in levels of training, and demonstrates how dissatisfactory the experience can feel to autistic parents.

## Discussion

This is the first study to look specifically at the experiences of autistic parents regarding interacting with statutory services on behalf of their autistic children, rather than at the experiences of non-autistic parents of autistic children. While some themes were in keeping with themes that have arisen with non-autistic parents, some are experienced differently while others are unique to autistic parents.

The first superordinate theme ‘the wider system is the problem’ presented the ways participants felt generally dismissed and unsupported by services. This theme is also evident in previous research whereby non-autistic parents felt that educational providers ‘do not listen’ (Sproston et al., 2017), healthcare professionals failed to recognise the child’s differences and were unempathetic (Celia et al., 2020), and in a subtheme exploring more general experiences of autistic parents Marriott et al. (2022) identified a failure to recognise or provide validation for the increased complexity of being an autistic parent of an autistic child. In contrast to previous findings, participants in this study were very conscious to clarify it was not the individual professionals that were unhelpful, but the wider system in which they were working that was unfit for purpose. Participants demonstrated empathy for how difficult working in these systems might be and cited several instances where individual professionals had made a difference through their understanding and compassion. Nevertheless, the overall feeling that there was a lack of support was an important factor in reduced wellbeing and parental stress (Burke & Hodapp, 2014).

One frustration raised in relation to the systems was a sense of injustice. Characteristics of autism such as ‘inflexible adherence to routines’ and ‘rigid thinking patterns’ (American Psychiatric Association, 2013) may contribute to an increased need for fairness and authenticity among autistic people (Kirchner et al., 2016), and might contribute to autistic parents particularly struggling when professionals and services are unable to act consistently or to adhere with legislation. Participants also talked about an imbalance in the system in terms of certain families being at an advantage due to having more resources such as time, money and education. This is in keeping with previous research where the issue of lower socioeconomic status and lower educational status were found to be barriers to successful advocacy (Boshoff et al., 2016).

These difficulties faced within the wider system meant that participants felt that they needed to fight for the right support, which has been noted in previous studies as an important element of the experiences of non-autistic parents (Celia et al., 2020; Marriott et al., 2022; Sedgewick et al., 2018; Sproston et al., 2017) and so it is no surprise it would also arise as an important theme for autistic parents. Participants talked about the impact engaging in this fight had on their mental health, which is in keeping with previous reports from autistic parents (Marriott et al., 2022).

For some participants, this fight for support involved healthcare services, while for others it involved education. Previous studies exploring the experiences of non-autistic parents have found the quality of the relationship between parents and school has a direct impact on levels of stress where positive relationships resulted in parents experiencing significantly less stress (Burke & Hodapp, 2014). For these participants, the increased levels of stress they experienced for having to fight for the right support had a wide-reaching impact on their mental health. Conversely, where participants reported experiencing understanding and compassion from professionals who had their own personal experience, it went a long way to reducing their stress levels.

The second superordinate theme ‘feeling judged and stigmatised’ is one particularly relevant to autistic parents of autistic children, and has not come up in previous research into the experiences of non-autistic parents although it has come up in research examining experiences of autistic adults (Cage et al., 2018; Perry et al., 2022). All participants indicated a preference for using the term ‘autistic’ and expressed frustration over professionals using terms such as ‘Autistic Spectrum Disorder’, which feels stigmatising and ‘high functioning’ that implies a linear conceptualisation of autism. Such terms can either strengthen negative perceptions of autism or be misleading in terms of the individual’s needs. This raises the important point that it is good practice to ask an autistic person what terminology and language is most meaningful to them, as what may be acceptable to one autistic person, may be interpreted as offensive to another.

Many participants felt one solution was improved training for professionals, judging existing training as generally outdated and focused on the medical model of autism rather than the social model, leaving parents in the position of feeling the need to rebel against this understanding and to educate professionals, while also advocating for their children. It is not uncommon for individuals to feel more knowledgeable than the professionals (Griffith et al., 2012; Mason et al., 2021). The need for additional training for healthcare providers in providing adequate support for autistic patients has also been highlighted in previous research, especially in the ability to recognise that adult patients may be autistic (Zerbo et al., 2015).

This lack of understanding for some resulted in a reluctance to disclose their own diagnosis for fear of being ‘othered’ and deemed as being unable to be good parents due to having a ‘disability’ (Bertilsdotter Rosqvist & Lövgren, 2013). Camouflaging or masking one’s autistic identity is not an uncommon strategy to gain acceptance (Cage et al., 2018), and many participants felt safer in their interactions with professionals by avoiding disclosing their own diagnosis. This is in keeping with the findings of Thompson-Hodgetts et al. (2020) that the lived experience of autistic adults was generally negative when disclosing their diagnosis to others, especially where there was a feeling of being unsupported and there was a lack of trust, despite the best intentions of those being disclosed to evidenced in analogue research. Conversely, some of the participants’ reported disclosure was empowering as they felt more credible when advocating for the needs of their children. Indeed, previous research has indicated where professionals have a good understanding of autism, autistic parents were more likely to receive increased respect (Dugdale et al., 2021). This split in how comfortable participants felt to disclose their own diagnosis, appeared linked to previous experience, and this indicates that there is likely to be variability in the understanding of autism among professionals.

On the whole, the findings from this study are in line with the SPACE framework (Doherty et al., 2023). In particular, the recommendations for predictability, where services follow through on what is planned, and acceptance and empathy in relation to the disclosure of diagnosis are needs represented in the experience of participants.

### Reflections

Most experiences covered by participants felt very familiar. That half of the participants had experienced increased credibility and felt empowered by disclosing their own diagnosis was in keeping with personal experience. However, it was both surprising and disappointing to hear that for many, disclosure had not been a positive experience.

### Future directions

The findings from this study indicate several considerations to be taken into account when supporting autistic parents seeking support for their autistic children. Due to the experiences of participants feeling stigmatised and misunderstood it is recommended that professionals consider the possibility parents of autistic children may also be autistic and ask families what terminology they prefer to use. It is also recommended that all training be co-developed with and delivered by autistic adults and provided to all professionals who may encounter autistic families. It is hoped that the involvement of autistic adults in the running of training would encourage a strength-based understanding of autism, rather than a deficit-based understanding, and allow the autistic community to share with professionals the information they feel would be the most helpful to aid understanding. The provision of support groups is also recommended to help lessen the negative impact on the wellbeing of these parents.

### Limitations

The sample used in this study consisted only of women with a mean age of 46, who were largely White British and well educated, and may not be transferable to the wider autistic population. There was no representation of autistic fathers, and no representation of younger parents, which would be beneficial to explore in future research. It is also important to note this study only included individuals who had received an autism diagnosis, which may have excluded autistic parents struggling to get a diagnosis for various reasons. All participants had also received their diagnosis in adulthood and so may not be representative of the experiences and views of autistic parents diagnosed as children.

## Conclusion

The findings of this unique IPA study highlight some very specific difficulties faced by autistic parents when advocating for their autistic children and improvements that would be beneficial in both healthcare and educational settings. Autistic parents have experienced feeling stigmatised, judged, dismissed and misunderstood. The experiences shared by the autistic mothers in this study about the struggles they have faced when interacting with statutory services for their autistic children highlighted the significant impact that these interactions can have on their overall wellbeing and experience of parenthood.
